# Editorial: Protein Aggregation and Solubility in Microorganisms (Archaea, Bacteria and Unicellular Eukaryotes): Implications and Applications

**DOI:** 10.3389/fmicb.2020.620239

**Published:** 2020-11-30

**Authors:** Georgios Skretas, Salvador Ventura

**Affiliations:** ^1^Institute of Chemical Biology, National Hellenic Research Foundation, Athens, Greece; ^2^Departament de Bioquimica i Biologia Molecular, Institut de Biotecnologia i Biomedicina, Universitat Autònoma de Barcelona, Barcelona, Spain

**Keywords:** protein folding, protein solubility, protein aggregation, recombinant protein production, microorganisms, pathogenic amyloids, functional amyloids

Proteins constitute the functional molecules of life. Typically, only folded and soluble proteins are functional, whereas the failure of a protein to adopt its intended three-dimensional structure (protein misfolding) usually results in the formation of biologically inactive, insoluble protein aggregates. Thus, protein solubility is critical for the maintenance of healthy cellular physiology and a major concern for both basic biomedical research and the biotechnology industry. At the same time, protein misfolding and aggregation have emerged as common pathological features of more than 50 human diseases, which can result in neurodegeneration, metabolic dysfunction, heart and kidney failure, blindness, and other maladies (Chiti and Dobson, 2017).

When isolated recombinant proteins are needed for biochemical/structural studies, and as industrial bioproducts, microbial cell hosts are typically the first choice as protein production factories (Makino et al., 2011a). Their industrial importance is well-reflected by the fact that approximately half of all approved recombinant biopharmaceuticals are produced in unicellular hosts, such as *Escherichia coli* and *Saccharomyces cerevisiae* (Ferrer-Miralles et al., 2009). In this Research Topic, Kang and Seong describe how microbial hosts can be used to produce antibody fragments, a special and important class of biotherapeutic molecules (Kang and Seong). Indeed, six of the currently marketed antibody fragment pharmaceuticals, either in the single-chain variable fragment (scFv) or the fragment antigen binding (Fab) format, are produced in *E. coli*. Unfortunately, a significant proportion of antibody fragment sequences suffer from low solubility, low stability, or high-aggregation propensity. The authors present a thorough review of currently utilized strategies for addressing these issues in microbial expression hosts. These include protein engineering approaches, such as rational mutagenesis, computational design, directed evolution, and use of solubility-enhancing fusion partners, as well as cell host engineering techniques, primarily through molecular-chaperone co-expression. Since microbial hosts can also be used to produce and to engineer full-length monoclonal antibodies (Mazor et al., 2007; Makino et al., 2011b), it is conceivable that the list of microbially produced marketed biopharmaceuticals could soon be expanded to include these molecules as well.

Upon production in microbial factories, many recombinant polypeptides undergo irregular or incomplete folding processes that usually result in their accumulation as insoluble aggregates, also known as inclusion bodies (IBs). Until very recently, this phenomenon was perceived merely as an unwanted side-effect of recombinant production, and IBs as a kind of useless “dust balls.” The quite surprising observation, however, that certain microbial aggregates can contain a fraction of molecules with native-like structure, which can retain biological activity, provided the motivation to exploit this initially unwanted phenomenon for understanding the relationship between conformational protein quality and solubility, as well as for the development of useful new applications (Garcia-Fruitos et al., 2005; de Marco et al., 2019). Based on these, Panda and co-workers set out to investigate the effect of varying the expression temperature on the formation, the morphology and the biological activity of bacterial IBs Singh et al.. By using the *E. coli* asparaginase II, a tetrameric enzyme, which can retain enzymic activity in inclusion body form, they have made a quite unanticipated observation: the specific catalytic activity of asparaginase inclusion bodies increases as the production temperatures increases. This observation contradicts previous studies with other model proteins, where the opposite effects were noted, and suggests that the relationship between solubility, aggregation, and conformational quality may be protein specific.

An additional exciting property of proteins embedded in microbial deposits is that they resemble the amyloid fibrils formed in several human neurodegenerative and other protein aggregation diseases (Wang et al., 2008). This feature makes microbial cells appropriate model systems for studying the mechanisms of pathogenic protein aggregation in an environment that is very simple and, at the same time, more biologically relevant than a test tube (Dasari et al., 2011; Villar-Pique and Ventura, 2012). Furthermore, it can allow the rapid screening of therapeutic drug candidates (Villar-Pique et al., 2012). In some instances, microbes can be even engineered to autonomously perform both the production of the molecular libraries under investigation and their screening for aggregation-inhibitory activity (Matis et al., 2017; Delivoria et al., 2019). Yeast cells, in particular, are also excellent systems for conveniently studying the link between protein aggregation and cytotoxicity (Kostelidou et al., 2018). In this direction, Regina Menezes' lab used *Saccharomyces cerevisiae* as a model organism to study the properties of different forms of the islet amyloid polypeptide (IAPP), a protein whose aggregation can be toxic for pancreatic β-cells and contribute to the pathology of type 2 diabetes Raimundo et al.. IAPP, also known as amylin, is a polypeptide whose mature form is produced as a result of the sequential proteolytic processing of the initial pre-pro-IAPP sequence. Raimundo et al. used yeast cells to investigate the aggregation and subsequent cytotoxicity of premature forms of IAPP compared to its mature form. These investigators have found that the initial precursor polypeptide pre-pro-IAPP is particularly toxic and that this toxicity is associated with the intracellular accumulation of insoluble oligomeric species. These observations point to a role of premature IAPP forms in the etiology of type 2 diabetes.

Protein aggregation is a hallmark of many diseases; however, it is becoming increasingly evident that living systems -and especially microorganisms- can exploit the phenomenon of protein aggregation and amyloid formation for their own benefit. This type of amyloid aggregates is generally referred to as “functional amyloids” (Otzen and Riek, 2019). One such example of functional amyloid is the CsgA protein of *E. coli*, whose regulated aggregation leads to amyloid formation and enables this enterobacterium to form highly rigid and resistant extracellular biofilms. These biofilms endow *E. coli* colonies with a very effective defense mechanism against external attacks, such as antibiotic treatment. Thus, the aggregation of CsgA and other similar microbial proteins are major targets for antibiotic discovery. In an attempt to provide an easy-to-work-with starting point for the screening of chemical or biological agents that inhibit CsgA aggregation and biofilm formation, Matthew Chapman and his colleagues engineered a double cysteine variant of the protein, termed CsgA_CC_, where the Cys residues have been introduced into a region of the protein that is critical for amyloid formation (Balistreri et al.). Under oxidizing conditions, CsgA_CC_ was shown to adopt a conformation that contains a non-native disulfide, which prevents aggregation of the protein. Upon addition of a reducing agent, however, this covalent bond is broken and CsgA proceeds to a native-like aggregation pathway, resulting in amyloid aggregates, which are virtually indistinguishable from those of the wild-type protein. CsgA sequence is highly aggregation-prone and not easy to handle, as it is constantly changing its polymerization state. In contrast, in CsgA_CC_, the aggregation reaction can be controlled chemically, offering a convenient starting point for screening expeditions looking for CsgA aggregation inhibitors.

Due to their central role in biology, protein folding, and solubility are controlled genetically, transcriptionally, and at the protein sequence and structural level. Besides, a well-conserved cellular machinery assists the folding of polypeptides and preserves proteostasis. Molecular chaperones, such as the ones belonging to the heat shock protein 70 (Hsp70) and Hsp90 families play a central role in the maintenance of proper protein folding, solubility, and, if needed, controlled clearance. To assist these major cellular folding factors and for specialized missions, cells also use the so-called small heat shock proteins (Hsps). One example of them is Hsp33, a redox state-regulated molecular chaperone that promotes bacterial and algae survival under heat and oxidative stress conditions. The laboratory of Dana Reichmann has applied bioinformatic analyses to identify new Hsp33 candidates and have found that, apart from bacteria and algae, similar sequences can be found in eukaryotic organisms, mainly in unicellular parasites and organisms residing in extreme environments (Aramin et al.). Based on this observation, they investigated whether Hsp33 could be an essential factor for the survival of these eukaryotic microbes under harsh living conditions. They chose to study the Hsp33 protein from *Trypanosoma brucei*, a eukaryotic pathogen causing African sleeping sickness. They called this protein TrypOx and investigated whether this parasite uses this factor to prevent its proteins from misfolding and as a defensive strategy against the oxidative stress attack exerted by the immune system of the infected host, in this case human cells. Aramin et al. have found that TrypOx is indeed a redox sensitive protein that, through interaction with the main Hsp70/Hsp40 chaperone complex, assists protein folding and contributes to *T. brucei* survival under oxidative stress. Thus, TrypOx and, by extension, the Hsp33 proteins from other eukaryotic pathogens may be relevant targets for drug discovery.

Overall, the study of protein folding, solubility, and aggregation in all types of microorganisms (archaea, bacteria, and unicellular eukaryotes) is an exciting and increasingly vibrant field that can provide both important insights in basic science as well as new tools for drug discovery/development and other important biotechnological applications.

## Author Contributions

GS and SV wrote the paper. All authors have read and approved the final version of the manuscript.

## Conflict of Interest

The authors declare that the research was conducted in the absence of any commercial or financial relationships that could be construed as a potential conflict of interest.
